# Biopsy pathology of the breast second edition

**DOI:** 10.1038/sj.bjc.6600586

**Published:** 2002-10-21

**Authors:** John P Sloane

**Affiliations:** University of Leicester, UK

## Abstract

*British Journal of Cancer* (2002) **87**, 1055–1055. doi:10.1038/sj.bjc.6600586 www.bjcancer.com

© 2002 Cancer Research UK

## 

The first edition of Biopsy Pathology of the Breast appeared in 1985. Between then and 2000, which is when John Sloane completed his edition before his untimely death, things have changed considerably in the field of breast pathology. Mammographic screening is now used extensively with national programmes, which has brought about changes in practice and in the nature of the lesions identified. Pathologists play important roles in the decision making processes of the multidisciplinary team meetings.

The second edition of Biopsy Pathology certainly reflects these developments. The number of chapters has expanded, and are now quite diverse. Pathological examination includes a clear description of dealing with mammographically detected and localised lesions. Minimum data sets and quality assurance are considered under the section on reporting. The chapter on histochemical methods has been extended to include immunohistochemistry and molecule technique. This is a mixture of markers of value in diagnosis, e.g. basement membrane, receptors, and research orientated markers. Non-operative diagnosis includes a section on core biopsy, with criteria used in screening. The section on non-neoplastic epithelial changes now includes detailed descriptions of radial scar/complex sclerosing lesions with useful comments.

A problem area that gets most people consulting the books (and checking definitions) is that of atypical hyperpasia. There is a detailed description of the problems in diagnosing this lesion, and the molecular changes, but more illustrations would have been of value. The chapter on ductal carcinoma *in situ* has a detailed review of the various classification systems, and covers rarer types, pathological features relating to recurrence and molecular alterations. The chapter on lobular *in situ* neoplasia presents current thinking. Premalignancy in the breast has similarly been up dated to include molecular studies.

All of the common and rarer types of invasive carcinomas are described with comments about the important pathological and behavioural features, including recent data such as cancers occurring in patients with inherited syndromes. Morphological and molecular features of prognostic significance are covered separately.

The chapter on fibroadenoma/phyllodes tumours has been expanded and updated. The rest of the book deals with miscellaneous lesions.

The illustrations are a mixture of black and white and colour. The latter are generally of a better standard. In places more figures would be of benefit for the pathologist trying to sort out a problem or ensure that he/she is following the guidelines. In this sense it is not really a ‘biopsy’ pathology book since it is a book to read to find out about recent advances and thinking rather than as a bench book to sort out problems. It could therefore be of value to a wide range of people – those in training, researchers wanting a breadth of information, pathologists – but not necessarily those wanting to solve that diagnostic problem. Hopefully it will be kept updated by those who have undertaken the editing of this book which contains so much of the experience and views of the late John Sloane.

